# *In Vitro* Bactericidal Activity of 4- and 5-Chloro-2-hydroxy-*N*-[1-oxo-1-(phenylamino)alkan-2-yl]benzamides against MRSA

**DOI:** 10.1155/2015/349534

**Published:** 2015-01-15

**Authors:** Iveta Zadrazilova, Sarka Pospisilova, Karel Pauk, Ales Imramovsky, Jarmila Vinsova, Alois Cizek, Josef Jampilek

**Affiliations:** ^1^Department of Chemical Drugs, Faculty of Pharmacy, University of Veterinary and Pharmaceutical Sciences Brno, Palackeho 1/3, 612 42 Brno, Czech Republic; ^2^Department of Infectious Diseases and Microbiology, Faculty of Veterinary Medicine, University of Veterinary and Pharmaceutical Sciences Brno, Palackeho 1/3, 612 42 Brno, Czech Republic; ^3^CEITEC VFU, University of Veterinary and Pharmaceutical Sciences Brno, Palackeho 1/3, 612 42 Brno, Czech Republic; ^4^Institute of Organic Chemistry and Technology, Faculty of Chemical Technology, University of Pardubice, Studentska 573, 532 10 Pardubice, Czech Republic; ^5^Department of Inorganic and Organic Chemistry, Faculty of Pharmacy in Hradec Kralove, Charles University in Prague, Heyrovskeho 1203, 500 05 Hradec Kralove, Czech Republic

## Abstract

A series of nine substituted 2-hydroxy-*N*-[1-oxo-1-(phenylamino)alkan-2-yl]benzamides was assessed as prospective bactericidal agents against three clinical isolates of methicillin-resistant *Staphylococcus aureus* (MRSA) and *S. aureus* ATCC 29213 as the reference and quality control strain. The minimum bactericidal concentration was determined by subculturing aliquots from MIC determination onto substance-free agar plates. The bactericidal kinetics of compounds 5-chloro-2-hydroxy-*N*-[(2*S*)-3-methyl-1-oxo-1-{[4-(trifluoromethyl)phenyl]amino}butan-2-yl]benzamide (**1f**), *N*-{(2*S*)-1-[(4-bromophenyl)amino]-3-methyl-1-oxobutan-2-yl}-4-chloro-2-hydroxybenzamide (**1g**), and 4-chloro-*N*-{(2*S*)-1-[(3,4-dichlorophenyl)amino]-3-methyl-1-oxobutan-2-yl}-2-hydroxybenzamide (**1h**) was established by time-kill assay with a final concentration of the compound equal to 1x, 2x, and 4x MIC; aliquots were removed at 0, 4, 6, 8, and 24 h time points. The most potent bactericidal agent was compound **1f** exhibiting remarkable rapid concentration-dependent bactericidal effect even at 2x MIC at 4, 6, and 8 h (with a reduction in bacterial count ranging from 3.08 to 3.75 log_10_ CFU/mL) and at 4x MIC at 4, 6, 8, and 24 h (5.30 log_10_ CFU/mL reduction in bacterial count) after incubation against MRSA 63718. Reliable bactericidal effect against other strains was maintained at 4x MIC at 24 h.

## 1. Introduction

The antibiotic resistance of invasive pathogens has become one of the most challenging and persistent health problems [1]. Methicillin-resistant* Staphylococcus aureus* (MRSA) has become the most common clinically relevant multiresistant pathogen [2] causing both healthcare-associated and community-acquired bloodstream infections with mortality rates up to 40% [3].

The prevalence of MRSA is increasing worldwide and, according to the latest information of the European Centre for Disease Prevention and Control from 2012 [4], can be considered alarming in some European countries, especially in Portugal and Romania, where ≥50% of all* S. aureus* isolates from invasive infections were identified as MRSA in 2012 (although, e.g., in Romania the prevalence of MRSA was 25–50% in 2010), followed by Italy, Greece, and Poland with 25–50% isolates being MRSA in 2012 (for comparison, in Poland MRSA isolates constituted 10–25% from all* S. aureus* isolates in 2010).

The treatment failure of vancomycin, the therapeutic anti-MRSA agent of choice, due to the strains with elevated vancomycin minimum inhibitory concentration (MIC) values (i.e., the lowest concentration of an antimicrobial that will inhibit the visible growth of a microorganism) within the susceptible range was described previously [5, 6]. Thus, the emergence of MRSA (and vancomycin-resistant* S. aureus *in the recent years as well [7]) makes the discovery of new molecular scaffolds a priority, and the current situation even necessitates the reengineering and repositioning of some old drug families to achieve adequate control of these bacteria [8]. However, for the treatment of* S. aureus* bloodstream infections, bactericidal antimicrobial agents are considered to be superior to bacteriostatic drugs [9]. This fact should be considered during the development of effective and safe treatment options for MRSA infections.

The history of clinical usage of salicylanilides (2-hydroxy-*N*-phenylbenzamides) dates back to the 1940s in therapy of tinea capitis, followed by the discovery of their anthelmintic properties in the mid 1950s [10]. Nowadays, salicylanilides (SALs) are a class of aromatic compounds possessing a wide range of interesting pharmacological activities, such as anthelmintic [11], antibacterial [12, 13], antimycobacterial [13], antifungal [14], and antiviral [15, 16], among others. Despite being studied since the 1960s, the mechanism of action responsible for biological activities of these compounds has not been explained so far. SALs have been found to inhibit the two-component regulatory systems (TCS) of bacteria [17]. The latest studies specified them also as selective inhibitors of interleukin-12p40 production that plays a specific role in initiation, expansion, and control of cellular response to tuberculosis [18]. Furthermore, salicylanilides have been recognised as inhibitors of some bacterial enzymes, such as sortase A from* S. aureus* [19], d-alanine-d-alanine ligase [20], or transglycosylases from* S. aureus* (but not from* M. tuberculosis*) [12]. These enzymes participate in secretion of various proteins or in biosynthesis of bacterial cell wall. Recently, salicylanilides-like derivatives were described to inhibit two enzymes essential for mycobacteria: (i) methionine aminopeptidase, catalyzing a key step of the posttranslational modification of nascent proteins, and (ii) isocitrate lyase, which is essential for the metabolism of fatty acids [21]. Thus, SALs seem to be promising candidates for development of new antibacterial agents with a novel mechanism of action. Such new agents could be a solution to the resistance challenges.

This study is a follow-up paper to a recently published article [13]. The synthesis of the series of novel derivatives of salicylamides, 4- and 5-chloro-2-hydroxy-*N*-[1-oxo-1-(phenylamino)alkan-2-yl]benzamides, called diamides due to their skeleton (for general structure see Table 1), was described previously [13, 22], and their antimycobacterial and antibacterial activities against various bacterial species were reported [13]. As these compounds expressed very significant antibacterial activity with low MIC values against clinical isolates of MRSA as representatives of multidrug-resistant bacteria, we decided to extend the knowledge about the antibacterial properties of these compounds against MRSA.

The aim of the current study was to assess the overall* in vitro* bactericidal activity of nine newly synthesized diamides in dependence on time and concentration against clinical isolates of MRSA as representatives of multidrug-resistant bacteria. To the best of our knowledge, this is the first study dealing with the evaluation of novel microbiological characteristics of SAL analogues and revealing their bactericidal effect.

## 2. Materials and Methods

### 2.1. Synthesis of Compounds

The synthetic pathway of the series of novel diamides was described recently [13, 22], and their structures (see Table 1) were confirmed by IR, NMR, and MS spectrometry, and the purity of the compounds was checked by CHN analysis [13, 22].

### 2.2. Culture Media and Antibiotics

All media were prepared from dehydrated powders (Oxoid, Basingstoke, UK) according to manufacturer's instructions. Ampicillin (AMP), ciprofloxacin (CPX), and vancomycin (VAN) were obtained from Sigma-Aldrich (St. Louis, MO, USA). Stock solutions were prepared by dissolving the antibiotic in sterile deionized water [26].

### 2.3. Bacterial Strains

*In vitro* antibacterial activity of the synthesized compounds was evaluated against representatives of multidrug-resistant bacteria, three clinical isolates of MRSA: clinical isolate of animal origin MRSA 63718 (Department of Infectious Diseases and Microbiology, Faculty of Veterinary Medicine, University of Veterinary and Pharmaceutical Sciences Brno, Czech Republic) carrying* mecA* gene; MRSA SA 630 [27]; and MRSA SA 3202 [27] (National Institute of Public Health, Prague, Czech Republic) both of human origin. Suspected colonies were confirmed by PCR; a 108 bp fragment specific for* S. aureus* was detected [28]. All isolates were tested for the presence of the* mecA* gene encoding methicillin resistance [29]. These three clinical isolates were classified as vancomycin-susceptible (but with higher MIC of vancomycin equal to 2 *μ*g/mL (VA2-MRSA) within the susceptible range for MRSA 63718) methicillin-resistant* S. aureus* (VS-MRSA). For the MICs of vancomycin, see Table 1. Vancomycin-susceptible methicillin-susceptible* Staphylococcus aureus *(VS-MSSA) ATCC 29213, obtained from the American Type Culture Collection, was used as the reference and quality control strain. The bacteria were stored at −80°C and were kept on blood agar plates (Columbia agar base with 5% ovine blood) between experiments.

### 2.4. Determination of Minimum Bactericidal Concentrations (MBCs)

The MBCs (i.e., the lowest concentrations of antibacterial agents required to kill a particular bacterium) were determined by subculturing aliquots (20 *μ*L) from wells with no visible bacterial growth and from control wells of MIC determination onto substance-free Mueller-Hinton agar (MHA) plates. The plates were incubated aerobically at 37°C for 24 h for colony count. The MBC was defined as the lowest concentration of substance, which produced ≥99.9% killing after 24 h of incubation as compared to the colony count of the starting inoculum [30]. To ensure reproducibility, each MBC assay was performed in at least triplicate on separate occasions.

### 2.5. Time-Kill Assays

Time-kill assays were performed by the broth macrodilution method according to previously described methodology [30] with some modifications. Briefly, flasks containing sterile fresh Mueller-Hinton broth (MHB) with the appropriate antimicrobial agent were inoculated with the test organism in logarithmic growth phase to obtain the starting inoculum with the concentration of approximately 7.5 × 10^6^ CFU/mL (actual inoculum concentrations ranged from 0.9 × 10^5^ to 2.9 × 10^6^ CFU/mL) and a final concentration of the antibiotic equal to 1x, 2x, and 4x MIC in 10 mL volume. For the determination of viable counts, aliquots were removed at 0, 4, 6, 8, and 24 h time points after inoculation, serially diluted in sterile phosphate buffered saline, and aliquots (20 *μ*L) were plated on MHA plates in duplicate. Colony counts were performed on plates yielding 6 to 60 colonies, and the mean was calculated. Antimicrobial carry-over was controlled by dilution and visual inspection of the distribution of colonies on the plates with observation of possible inhibition of growth at the site of the initial streaks. The plates were incubated at 37°C for 24 to 48 h, and the number of colonies was determined. To ensure reproducibility, each time-kill experiment was carried out in duplicate on separate occasions with results presented as the mean of all experiments. The growth control without the addition of antimicrobial agents and the control containing DMSO without any antimicrobial agent to exclude antibacterial activity of this solvent were included. Time-kill curves were constructed by plotting the log_10_ CFU per millilitre versus time (over 24 h), and the change in bacterial concentration was determined. The results were analysed by evaluating the numbers of strains that yielded Δ(log⁡_10_ CFU/mL) values of −1 (corresponding to 90% killing), −2 (99% killing), and −3 (99.9% killing) at 4, 6, 8, and 24 h compared to counts at 0 h. Bactericidal activity was defined as a reduction of at least 99.9% (≥3 log⁡_10_) of the total count of CFU/mL in the original inoculum.

## 3. Results and Discussion

Diamides seem to be promising candidates for antibacterial agents with very strong anti-MRSA activity, as it was published recently [13]. In the present study the series of nine newly synthesized diamides was evaluated as prospective bactericidal agents against representatives of multidrug-resistant bacteria, three clinical isolates of MRSA, and* Staphylococcus aureus *ATCC 29213 (methicillin-susceptible) as the reference and quality control strain. Since SALs and their analogues are known as compounds with bacteriostatic effect [31], this is the first study where SAL-like compounds were considered as prospective bactericidal agents and the dependence of bactericidal effect of these compounds on time and concentration was evaluated. Thus, absolutely novel microbiological characteristics of these compounds were revealed in the present study.

Recently MIC values of diamides expressed as molar concentrations in *μ*mol/L were published [13]. To allow comparison with MBC values of the present study, MICs in *μ*g/mL were calculated and are recorded in Table 1 along with the activity of reference antibacterial drugs, ampicillin, ciprofloxacin, and vancomycin. Potential bactericidal activity of diamides was assessed using MBC assay [26]. MBC values of all tested compounds are recorded in Table 1 as well.

Based on the obtained results, all compounds assessed as active according to MIC values in our previous study (**1f**–**i**) showed low or moderate MBC values against all four strains. The MBC values of these compounds did not exceed the highest tested drug concentration and ranged from 1 to 16 *μ*g/mL. In all cases, there were comparable MBC values for the clinical isolates of MRSA and the* S. aureus* reference strain.

Bactericidal activity is defined as a ratio of MBC to MIC of ≤4 [32]. Comparison of the MIC and MBC values of the discussed compounds for each isolate indicates that the effect of diamides was bactericidal for all active compounds. Compound 4-chloro-*N*-{(2*S*)-1-[(3,4-dichlorophenyl)amino]-1-oxo-3-phenylpropan-2-yl}-2-hydroxybenzamide (**1i**) with bacteriostatic effect against clinical isolates of MRSA 63718 and MRSA SA 3202 was the only exception from this rule. In Table 1 bactericidal activity is expressed in bold.

As mentioned above, SALs are known to exhibit a bacteriostatic effect [31], so it was very interesting to discover that diamides possess bactericidal activity. The amide bond (–CONH–) can cause interactions with a variety of enzymes [33]; therefore the presence of two amide bonds could be responsible for the bactericidal effect of diamides against MRSA. The activity of SALs and their analogues results from multiple mechanisms, which are still under investigation; for example, it was found that SALs are capable of inhibiting transglycosylases in later stages of* S. aureus *(including MRSA) cell wall biosynthesis [12]. These enzymes catalyse the step prior to the transpeptidation in the peptidoglycan biosynthesis and are responsible for polymerization of lipid II, which occurs at the outer face of the membrane [12]. Since antibacterial agents targeting cell wall biosynthesis act as bactericidal agents [30, 34], the failure in the cell wall biosynthesis due to the inhibition of transglycosylases could be responsible for bactericidal activity of diamides against MRSA.

Based on these findings, antibacterial active diamides with bactericidal effect against all four tested strains as prospective bactericidal agents were chosen for subsequent time-kill curve studies to determine the real dependence of bactericidal effect on concentration over time.

Compounds 5-chloro-2-hydroxy-*N*-[(2*S*)-3-methyl-1-oxo-1-{[4-(trifluoromethyl)-phenyl]amino}butan-2-yl]benzamide (**1f**),* N*-{(2*S*)-1-[(4-bromophenyl)amino]-3-methyl-1-oxobutan-2-yl}-4-chloro-2-hydroxybenzamide (**1g**) and 4-chloro-*N*-{(2*S*)-1-[(3,4-dichlorophenyl)amino]-3-methyl-1-oxobutan-2-yl}-2-hydroxybenzamide (**1h**) were tested in time-kill studies at 1x, 2x, and 4x MIC against all MRSA isolates and the* S. aureus* reference strain. The antibacterial effect of DMSO [35] used as the solvent of the tested compounds was excluded in this assay, as time-kill curves of this solvent were identical or very similar to those of the growth control. The extent of bacterial killing was estimated by the number of these strains showing a decrease ranging from 1 to 3 log⁡_10_ CFU/mL in viable cell count at different times after incubation. A summary of these data is presented in Table 2. Based on these data it can be concluded that the bactericidal potency of tested diamides against all four strains decreased as follows:** 1f** >** 1h** >** 1g**. No bactericidal activity (i.e., ≥3 log⁡_10_ CFU/mL decrease) was observed at 1x MIC for any strain and time after incubation tested. At 4x MIC from the four strains, compounds** 1f**,** 1 g**, and** 1h **killed 2, 1, and 2 strains, respectively, at 8 h after incubation and 4, 2, and 2 strains, respectively, at 24 h after incubation.

The findings of time-kill studies for each of the four staphylococci strains at exposure to compounds** 1f**,** 1g**, and** 1h** are summarized in Table 3. Bactericidal activity (i.e., ≥3 log⁡_10_ CFU/mL decrease) is expressed in bold.

For compound** 1f** rapid concentration-dependent antibacterial effect was recorded against clinical isolate of MRSA 63718. Time was not the predictive factor influencing the antibacterial activity because log⁡_10_ differences in CFU/mL from the starting inoculum were the same for 4x MIC (with the highest efficiency with a reduction in bacterial count of 5.30 log⁡_10_ CFU/mL) or very similar for 2x MIC (with a moderate regrowth after 24 h causing a loss of bactericidal activity) over 24 h. The bactericidal effect was maintained even at 2x MIC at 4 h after incubation for this strain (reduction of 3.08 log⁡_10_ CFU/mL). For the remaining strains, clinical isolates of MRSA SA 630, MRSA SA 3202, and* S. aureus* ATCC 29213, reliable bactericidal effect was recorded at 4x MIC at 24 h after incubation for all these strains with a reduction in bacterial count of 3.22, 3.30, and 3.65 log⁡_10_ CFU/mL, respectively.

For compound** 1g** bactericidal effect against MRSA 63718 was noticed at 2x MIC at 6 and 8 h after incubation and at 4x MIC at 4, 6, and 8 h after incubation with a reduction in bacterial count ranging from 3.10 to 3.58 log⁡_10_ CFU/mL. The most effective killing was achieved at 6 h for both concentrations. As in the case of compound** 1f**, a regrowth was observed after 24 h after incubation. For the remaining isolates of MRSA, SA 630 and SA 3202, bactericidal effect occurred only at 4x MIC at 24 h after incubation with a reduction in bacterial count of 3.38 and 4.01 log⁡_10_ CFU/mL, respectively. The highest bactericidal effect was recorded for MRSA SA 3202 at 4x MIC at 24 h after incubation. A reduction consistent with bacteriostatic effect (0.03 to 2.37 log⁡_10_ CFU/mL) was observed at other concentrations over time for both isolates. No bactericidal effect was observed for the* S. aureus* reference strain; compound** 1g** demonstrated a pattern of bacteriostatic activity against this strain with a reduction in bacterial count ranging from 0.07 to 2.33 log⁡_10_ CFU/mL at 4x MIC over time. In other cases, a slight increase in bacterial counts (i.e., overgrowth) compared with the starting inoculum was observed with values ranging from 0.10 to 1.57 log⁡_10_ CFU/mL for this reference strain.

For compound** 1h** bactericidal effect against MRSA 63718 was maintained at 4x MIC at 6 and 8 h after incubation with a reduction in bacterial count of 3.54 and 3.31 log⁡_10_ CFU/mL, respectively. The same as for** 1g**, the most potent bactericidal effect was maintained at 6 h after incubation. Regrowth at 24 h after incubation causing a loss of bactericidal activity was recorded similarly as with previous compounds. The reason for regrowth of the test organism at 24 h in the experiment is unknown. Most probably, selection of resistant mutants is responsible for this phenomenon [30]; degradation of the drug in the growth medium is not assumed, as regrowth was not observed for any other tested strain. For MRSA SA 630 concentration-dependent killing was recorded at 4x MIC at 6, 8, and 24 h after incubation with log⁡_10_ differences in CFU/mL from the starting inoculum being very similar over time (ranging from 3.18 to 3.39 log⁡_10_ CFU/mL). For MRSA SA 3202 reliable bactericidal effect was maintained only at 4x MIC at 24 h after incubation with a reduction in bacterial count of 3.02 log⁡_10_ CFU/mL. As for compound** 1g**, bacteriostatic activity against* S. aureus* reference strain was observed with a reduction in bacterial count ranging from 0.34 to 2.62 log⁡_10_ CFU/mL at 2x and 4x MIC. Overgrowth (values ranging from 0.04 to 1.43 log⁡_10_ CFU/mL) was recorded at 1x MIC for this strain.

It is of note that in all staphylococci strains with similar MICs and MBCs for compounds** 1g** and** 1h** the responsiveness to antibacterial activity of these compounds varied with clinical strains of MRSA being effectively killed and the reference strain remaining unaffected at 4x MIC.

There is a discrepancy between bactericidal results of MBC assay compared with time-kill kinetics. This difference could be caused by comparing microtiter (MBC assay) to macrobroth (time-kill assay) dilutions [36]. Moreover, although time-kill assays are more labour intensive and time consuming than MBC assays, they are recognised to provide a greater degree of characterisation of the cell eradication potential of antibacterial agents [37].

Concerning antibacterial effect, it is not generally important if the antibacterial agent is also bactericidal at higher concentrations, because the inhibition of bacterial proliferation usually achieves a therapeutic effect; the patient's immune system is capable of coping with the infection then [34]. However, bactericidal therapy could produce a better treatment result by rapid reduction of the bacterial load [38]. Moreover, in the case of an immune system disorder (e.g., immunosuppressive therapy, AIDS patients, etc.) bactericidal agents are unequivocally indicated. Considering steadily escalating numbers of immunocompromised patients with endocarditis, meningitis, or osteomyelitis in recent years, it is necessary to achieve bacterial killing and broaden the spectrum of antimicrobial agents with bactericidal active compounds [30].

The clinical outcome of MRSA bacteraemia is significantly influenced by vancomycin MIC. Treatment failure exceeding 60% for* S. aureus* with vancomycin MIC of 4 *μ*g/mL resulted in the change of susceptibility breakpoint from 4 *μ*g/mL to 2 *μ*g/mL by the Clinical and Laboratory Standards Institute (CLSI) in 2006 [23] as well as by the US Food and Drug Administration (FDA) in 2008 [39]. It has been recommended that for infections caused by MRSA strains with elevated vancomycin MICs (2 *μ*g/mL), alternative therapy should be considered [40]. It is of note that based on time-kill assays in the present study, all tested diamides (particularly compound** 1f** exhibiting rapid bactericidal concentration-dependent effect even at 2x MIC) were most effective against isolate MRSA 63718, which is the strain with elevated vancomycin MIC of 2 *μ*g/mL. The activity against the remaining isolates with vancomycin MIC of 1 *μ*g/mL was lower.

Considering the emergence of decreasing vancomycin susceptibility of MRSA isolates and thus the therapeutic efficacy of vancomycin therapy, our aim was to determine the potential bactericidal role of novel antibacterial compounds against MRSA* in vitro*. Based on the obtained results, diamides can be suitable candidates for such novel bactericidal active compounds presenting a promising starting point for further investigations to ascertain real* in vivo* activity and the exact mechanism of action.

## 4. Conclusions

The present study is the first evidence of bactericidal effect of SAL analogues. Compound 5-chloro-2-hydroxy-*N*-[(2*S*)-3-methyl-1-oxo-1-{[4-(trifluoromethyl)-phenyl]amino}butan-2-yl]benzamide (**1f**) exhibiting remarkable rapid concentration-dependent bactericidal effect at 2x MIC at 4, 6, and 8 h (with a reduction in bacterial count ranging from 3.08 to 3.75 log⁡_10_ CFU/mL) and at 4x MIC at 4, 6, 8, and 24 h (5.30 log⁡_10_ CFU/mL reduction in bacterial count) after incubation against MRSA 63718 was the most potent agent. Reliable bactericidal effect against other strains was maintained at 4x MIC at 24 h. For compounds* N*-{(2*S*)-1-[(4-bromophenyl)amino]-3-methyl-1-oxobutan-2-yl}-4-chloro-2-hydroxybenzamide (**1g**) and 4-chloro-*N*-{(2*S*)-1-[(3,4-dichlorophenyl)amino]-3-methyl-1-oxobutan-2-yl}-2-hydroxybenzamide (**1h**), a pattern of bacteriostatic effect was observed for* S. aureus* ATCC 29213, and the most potent bactericidal effect against MRSA 63718 was recorded at 4x MIC at 6 h after incubation for both compounds. Against other strains, reliable bactericidal effect was maintained at 4x MIC at 24 h after incubation. Considering the necessity to broaden the spectrum of bactericidal agents, diamides from the current study with a novel mechanism of action could present a very promising and interesting solution to this challenge for the future.

## Figures and Tables

**Table 1:** Chemical structures and *in vitro* MIC and MBC [*µ*g/mL] values of tested 5- and 4-chloro-2-hydroxy-*N*-[1-oxo-1-(phenylamino)alkan-2-yl]benzamides (bactericidal effect of individual compounds against particular strains marked in bold).

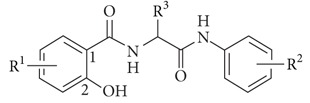											

Comp.	R^1^	R^2^	R^3^	MIC [*µ*g/mL]				MBC [*µ*g/mL]			
				1	2	3	4	1	2	3	4

**1a**	5-Cl	4-CH_3_	(*S*)-CH_3_	>256	>256	>256	>256	>256	>256	>256	>256
**1b**	5-Cl	4-CH_3_	(*S*)-CH(CH_3_)_2_	>256	>256	**32**	32	>256	>256	**128**	>256
**1c**	5-Cl	4-CH_3_	(*S*)-benzyl	>256	>256	>256	>256	>256	>256	>256	>256
**1d**	5-Cl	4-CH_3_	(*R*)-CH_2_-indolyl	>256	>256	>256	>256	>256	>256	>256	>256
**1e**	5-Cl	4-OCH_3_	(*S*)-CH(CH_3_)_2_	>256	>256	>256	>256	>256	>256	>256	>256
**1f**	5-Cl	4-CF_3_	(*S*)-CH(CH_3_)_2_	**4**	**2**	**2**	**2**	**4**	**4**	**8**	**4**
**1g**	4-Cl	4-Br	(*S*)-CH(CH_3_)_2_	**8**	**4**	**4**	**4**	**16**	**8**	**8**	**8**
**1h**	4-Cl	3,4-Cl	(*S*)-CH(CH_3_)_2_	**2**	**1**	**1**	**1**	**4**	**1**	**4**	**2**
**1i**	4-Cl	3,4-Cl	(*S*)-benzyl	1	**1**	0.5	**0.5**	8	**1**	8	**1**

**AMP**	—	—	—	>16	>16	>16	0.25	>16	>16	>16	0.25
**CPX**	—	—	—	>16	>16	>16	0.5	>16	>16	>16	0.5
**VAN**	—	—	—	2	1	1	1	2	1	1	1

Staphylococcal strains: 1: MRSA 63718; 2: MRSA SA 630; 3: MRSA SA 3202; 4: *Staphylococcus aureus* ATCC 29213.

AMP: ampicillin; CPX: ciprofloxacin; VAN: vancomycin.

MIC breakpoints for *S. aureus* ATCC 29213 [*µ*g/mL]: AMP > 2, CPX > 1, VAN > 2 [23–25].

**Table 2:** Extent of bacterial killing exerted by 5-chloro-2-hydroxy-*N*-[(2*S*)-3-methyl-1-oxo-1-{[4-(trifluoromethyl)-phenyl]amino}butan-2-yl]benzamide (**1f**), *N*-{(2*S*)-1-[(4-bromophenyl)amino]-3-methyl-1-oxobutan-2-yl}-4-chloro-2-hydroxybenzamide (**1g**), and 4-chloro-*N*-{(2*S*)-1-[(3,4-dichlorophenyl)amino]-3-methyl-1-oxobutan-2-yl}-2-hydroxybenzamide (**1h**) over time against four staphylococci strains.

Drug and concentration (multiplicity of MIC)	Number of strains showing the following log_10_ CFU/mL decrease^a^ at the designated incubation time											
	4 h			6 h			8 h			24 h		
	−1	−2	−3	−1	−2	−3	−1	−2	−3	−1	−2	−3
Comp. **1f**												
4× MIC	2	1	1	4	3	2	4	4	2	4	4	4
2× MIC	1	1	1	1	1	1	3	1	1	4	3	0
1× MIC	0	0	0	0	0	0	0	0	0	0	0	0
Comp. **1g**												
4× MIC	2	1	1	2	1	1	4	2	1	4	4	2
2× MIC	1	1	0	1	1	1	2	1	1	0	0	0
1× MIC	0	0	0	0	0	0	0	0	0	0	0	0
Comp. **1h**												
4× MIC	2	2	0	4	2	2	4	3	2	4	4	2
2× MIC	1	0	0	2	1	0	2	0	0	1	0	0
1× MIC	0	0	0	1	0	0	0	0	0	0	0	0

CFU: colony-forming units.

^a^Δ(log⁡_10_ CFU/mL) values of −1, −2, and − 3 log⁡_10_ CFU/mL correspond to 90% (bacteriostatic), 99% (bacteriostatic), and 99.9% (bactericidal) of killing, respectively.

**Table 3:** Change in viable counts (log_10_ CFU/mL) of MRSA and *S. aureus *strains following incubation for 24 h with 5-chloro-2-hydroxy-*N*-[(2*S*)-3-methyl-1-oxo-1-{[4-(trifluoromethyl)-phenyl]amino}butan-2-yl]benzamide (**1f**), *N*-{(2*S*)-1-[(4-bromophenyl)amino]-3-methyl-1-oxobutan-2-yl}-4-chloro-2-hydroxybenzamide (**1g**), and 4-chloro-*N*-{(2*S*)-1-[(3,4-dichlorophenyl)amino]-3-methyl-1-oxobutan-2-yl}-2-hydroxybenzamide (**1h**) (bactericidal effect is expressed in bold).

Strain	MIC/MBC	Conc.	Log_10_ difference in CFU/mL from inoculum			
			4 h	6 h	8 h	24 h
Comp. **1f**						
MRSA 63718	4/4	1× MIC	0.34	0.56	0.66	1.68
		2× MIC	−**3.08** ^a^	**−3.33**	**−3.75**	−2.40^b^
		4× MIC	**−5.30**	**−5.30**	**−5.30**	**−5.30**
MRSA SA 630	2/4	1× MIC	0.65	1.16	1.36	0.65
		2× MIC	−0.26	−0.77	−1.40	−2.07
		4× MIC	−0.83	**−3.26**	−2.52	**−3.22**
MRSA SA 3202	2/8	1× MIC	1.21	1.56	1.75	1.57
		2× MIC	0.06	−0.05	−0.65	−1.59
		4× MIC	−0.07	−1.05	−2.70	**−3.30**
*S.a. *	2/4	1× MIC	0.82	1.00	1.14	1.21
		2× MIC	−0.25	−0.74	−1.52	−2.52
		4× MIC	−1.17	−2.85	**−3.88**	**−3.65**
Comp. **1g**						
MRSA 63718	8/16	1× MIC	0.43	0.65	0.75	0.95
		2× MIC	−2.54	**−3.23**	**−3.15**	−0.76
		4× MIC	**−3.18**	**−3.58**	**−3.10**	−2.24
MRSA SA 630	4/8	1× MIC	0.98	1.42	1.57	0.55
		2× MIC	−0.12	−0.73	−1.50	−0.28
		4× MIC	−1.00	−1.54	−2.37	**−3.38**
MRSA SA 3202	4/8	1× MIC	0.56	1.47	1.70	2.14
		2× MIC	−0.03	−0.07	−0.82	−0.69
		4× MIC	−0.35	−0.56	−1.49	**−4.01**
*S.a. *	4/8	1× MIC	1.02	1.10	1.45	1.57
		2× MIC	0.16	0.10	0.80	−0.34
		4× MIC	−0.07	−0.11	−1.65	−2.33
Comp. **1h**						
MRSA 63718	2/4	1× MIC	−0.76	−1.09	−0.71	0.89
		2× MIC	−1.77	−2.07	−1.97	0.47
		4× MIC	−2.90	**−3.54**	**−3.31**	−2.65
MRSA SA 630	1/1	1× MIC	−0.27	−0.10	−0.09	1.42
		2× MIC	0.19	−1.19	−1.39	−0.30
		4× MIC	−2.72	**−3.21**	**−3.39**	**−3.18**
MRSA SA 3202	1/4	1× MIC	0.27	0.82	0.96	1.12
		2× MIC	0.17	−0.27	−0.53	−0.09
		4× MIC	−0.35	−1.13	−1.83	**−3.02**
*S.a. *	1/2	1× MIC	0.27	0.06	0.00	1.43
		2× MIC	0.04	−0.35	−0.94	−1.20
		4× MIC	−0.34	−1.29	−2.62	−2.61

CFU: colony-forming units; Conc.: concentration (multiplicity of MIC).

^a^ ≥3 log⁡_10_ reduction in CFU implies a bactericidal effect.

^b^ <3 log⁡_10_ reduction in CFU implies a bacteriostatic effect.
